# Identification of epithelial ciliated cells as the non‐endothelial source of mouse endocan

**DOI:** 10.1111/jcmm.18518

**Published:** 2024-07-03

**Authors:** Yoann Stordeur, Sophie Salomé Desnoulez, Patricia de Nadai, Joanne Balsamelli, Saliha Ait Yahia, Anne Tsicopoulos, Philippe Lassalle, Alexandre Gaudet

**Affiliations:** ^1^ Univ. Lille, CNRS, Inserm, CHU Lille Institut Pasteur de Lille, U1019 – UMR 9017 – CIIL – Center for Infection and Immunity of Lille Lille France; ^2^ CHU Lille, Service de Réanimation Pédiatrique Hôpital Jeanne de Flandres Lille France; ^3^ Univ. Lille, CNRS, Inserm, CHU Lille Institut Pasteur de Lille, US 41 – UAR 2014 – PLBS – BICeL Lille France; ^4^ Biothelis Lille France; ^5^ CHU Lille, Pôle de Médecine Intensive – Réanimation Hôpital Roger Salengro Lille France

Endocan is a circulating proteoglycan mostly secreted by pulmonary endothelial cells in human.^1^ Because of its immunomodulatory properties resulting from the inhibition of leukocyte diapedesis, endocan has been proposed as a candidate molecule for the treatment of acute respiratory distress syndrome (ARDS).^2^, ^3^ In vivo studies investigating the biological effects of endocan mostly rely on mouse models.^4^ However, major differences have been reported between the physiological properties of mouse and human endocan.^4^ One critical difference relies in the cellular populations responsible for the pulmonary expression of endocan. Moreover, it is still unknown whether measurement of blood endocan routinely performed for the daily clinical practice is a good surrogate for lung expression variations occurring in acute lung inflammation.

To address these questions, we first analysed online mouse transcriptomic databases (GSE108097—Mouse Cell Atlas)^5^ to identify candidate cell subsets involved in the pulmonary synthesis of mouse endocan through the expression of its mRNA *Esm1*.

We then used the RNAScope™ Multiplex Fluorescent Assay v2 (Bio‐Techne, Rennes, France), on formalin fixed paraffin embedded lung sections from 8 to 12 weeks old C57BL/6 male mice to detect the mRNA expression of *Esm1*, *Ccdc153*, a marker of epithelial ciliated cells, and *Tmem100*, a marker of endothelial cells.

Anaesthetised C57BL/6 mice were exposed to a tracheal instillation of either 2 mg/kg of lipopolysaccharide (LPS) diluted in 50 μL of phosphate‐buffered saline (PBS) to trigger a non‐lethal acute lung injury (ALI) model, or 50 μL of PBS without LPS in control mice, which were considered as the sham group, following a previously published model.^2^ We quantified the pulmonary expression of *Esm1* on whole sections at days 1, 3 and 5 following tracheal instillation of LPS using the RNAScope™ Multiplex Fluorescent Assay v2. *Ccdc153* and *Tmem100* were respectively used as epithelial and endothelial markers to characterize the source of expression of *Esm1*.

All images were acquired using a Zeiss LSM 880 confocal microscope, and processed using Imaris (Oxford instruments, Abingdon‐on‐Thames, UK). Magnification was set at 63X for the identification of *Esm1* pulmonary cellular source and at 40X for the quantification of *Esm1* expression in our ALI model.

Endocan blood levels were measured by ELISA (DIYEK M1®, Lunginnov, Lille, France) on serum after two centrifugations at 1500*g*.

Our analysis of publicly available transcriptomic data indicated that, in mouse, *Esm1* pulmonary expression was predominantly found in ciliated epithelial cells (Figure 1A, detailed list of clusters is shown in Table S1). We report the results regarding the identification and quantification by RNAscope™ of *Esm1* expression in non‐endothelial cells in Figure 1B,C. In naïve mice, we found an intense *Esm1* signal colocalizing with *Ccdc153*, along with a less intense *Esm1* signal colocalizing with *Tmem100* (Figure 1B). Quantification of *Esm1* in whole lung sections showed that endothelial cells are responsible for a majority of the total amount of expression of *Esm1*, with an epithelial/endothelial absolute expression ratio found between 0.24 and 0.41. However, the expression intensity, defined as absolute expression divided by surface area for each cell type, was higher for epithelial ciliated cells with an epithelial/endothelial expression intensity ratio found between 4.8 and 9.68 (Figure 1C). In other words, the expression of Esm1 in epithelial areas was 4.8–9.68 times more intense than in endothelial areas.

**FIGURE 1:**
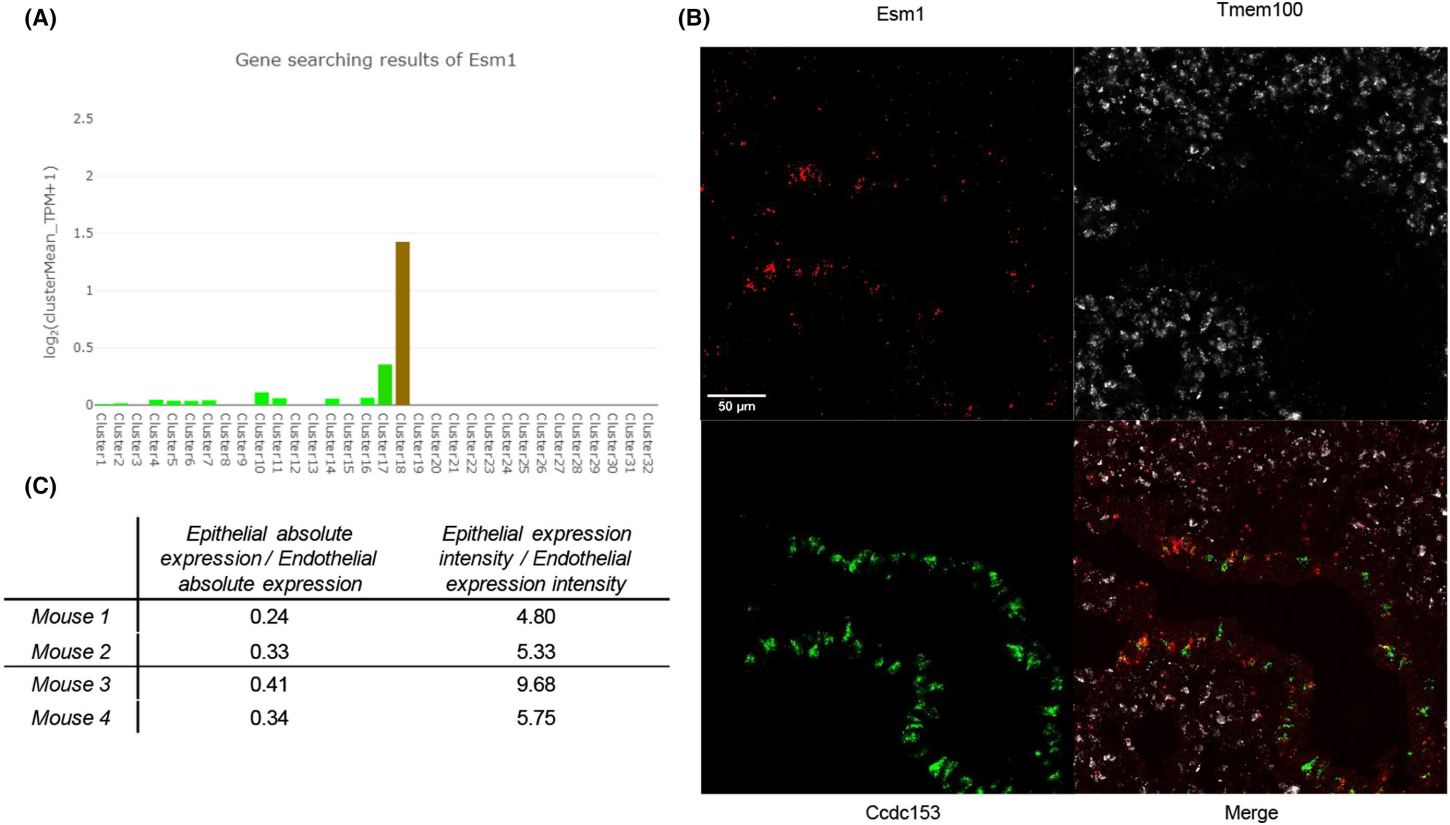
Identification of cell populations involved in the pulmonary synthesis of murine endocan. (A) Transcriptomic data analysis showing the repartition of pulmonary expression of *Esm1* according to cell type in C57BL/6 adult male mice. Predominant expression is found in Cluster 18, corresponding to ciliated epithelial cells (brown bar). The second most important source of expression is found in Cluster 17, corresponding to endothelial cells. Little expression of *Esm1* is found in other cell populations corresponding to fibroblasts and immunological cells. Mouse Cell Atlas (http://bis.zju.edu.cn/MCA/index.html). (B) RNAScope® staining in healthy C57BL/6 mouse lung section at 63X magnification. Staining was simultaneously performed for *Esm1* (red), *Tmem100* (white) and *Ccdc153* (green). (C) Ratios of lung *Esm1* absolute expression and expression intensity in epithelial cells over endothelial cells in healthy C57BL/6 mice. *Esm1* absolute expression was assessed on whole lung sections by quantifying the *Esm1* signal trough fluorescence intensity and attributing it to either epithelial expression if colocalized with *Ccdc153* signal or endothelial expression otherwise. Expression intensity was defined as the ratio of absolute expression over the surface of area of interest.

In our ALI model, the kinetics of *Esm1* expression surface showed no significant variation over time in LPS‐challenged mice versus control mice. However, we found a trend for a decrease from days 1 to 5 for the overall tissue (ϕ = 0.14) (Figure 2A–C). The epithelial/endothelial absolute expression and expression intensity ratios significantly increased over time in LPS‐challenged mice vs control mice (ϕ = 0.04 and ϕ = 0.003, respectively), especially at day 3 (*p* = 0.03) (Figure 2D,E). In other words, following LPS administration, the share of expression of *Esm1* taking its source from epithelial cells increased over time. In addition, these results suggest that this increase in the epithelial share of *Esm1* expression seemed notably explained by a stronger intensity of *Esm1* expression in epithelial areas relatively to endothelial areas. After LPS administration, blood levels of endocan reached their lowest level at day 1, at 0.68 ng/mL (0–1.93), then rose back to levels observed in the control group at day 5 (ϕ = 0.009) (Figure 2F). The kinetics of blood endocan and *Esm1* expression in lung had divergent patterns, as pulmonary expression still remained low by day 5. There was a negative correlation between endocan blood levels and the epithelial/endothelial intensity of expression ratio (*ρ* = −0.48, *p* = 0.008). As reflected by this result, the highest relative intensity of *Esm1* expression in epithelial areas and lowest relative intensity of *Esm1* expression in endothelial areas were observed in mice with the lowest levels of blood endocan (Figure 2G).

**FIGURE 2:**
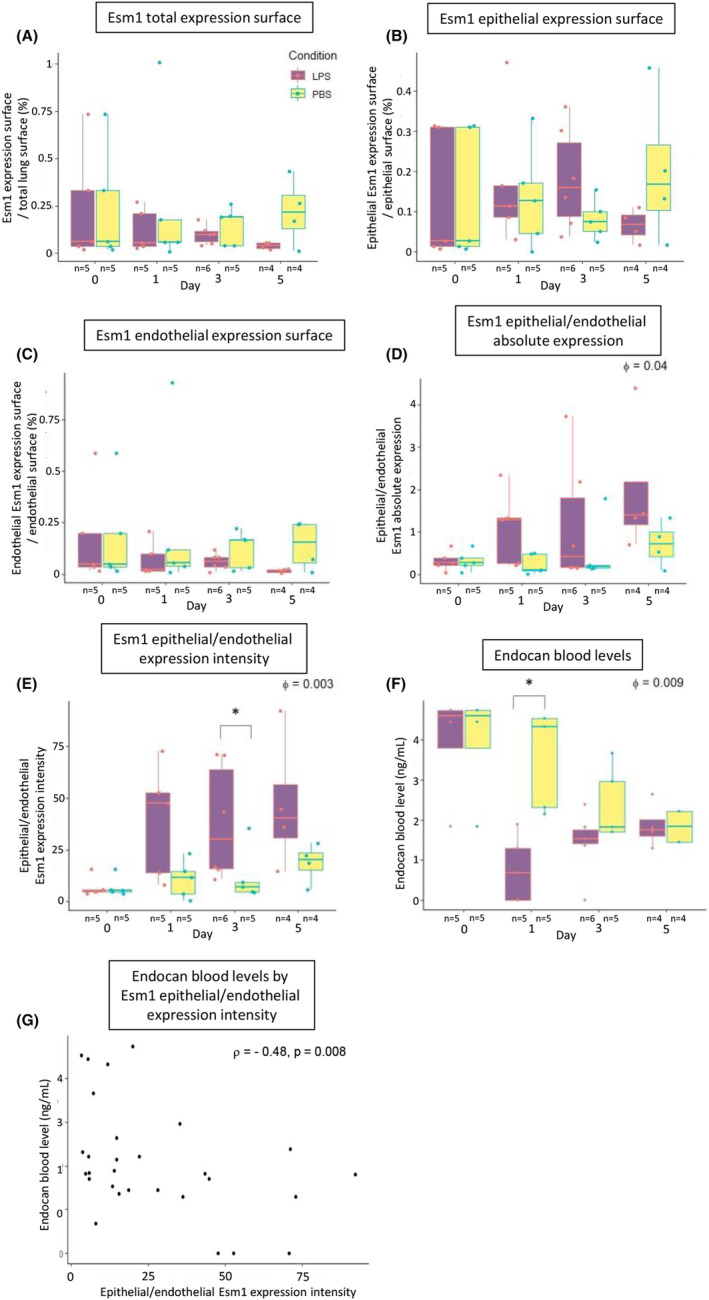
Variations of *Esm1* pulmonary expression and endocan blood levels during ALI. (A) Kinetics of *Esm1* expression surface over total lung surface assessed in whole lung sections collected at days 0, 1, 3 and 5 of ALI. (B) Kinetics of epithelial *Esm1* expression surface over total epithelial surface assessed in whole lung sections collected at days 0, 1, 3 and 5 of ALI. Epithelial area was defined as the area with predominant expression of *Ccdc153*. (C) Kinetics of endothelial *Esm1* expression surface over total endothelial surface assessed in whole lung sections collected at days 0, 1, 3 and 5 of ALI. Endothelial area was defined as the area with predominant expression of *Tmem100*. D) Kinetics of the ratio of *Esm1* absolute expression in epithelial cells over endothelial cells in whole lung sections collected at days 0, 1, 3 and 5 of ALI. A significantly higher ratio is observed in the LPS group (Φ = 0.04 by linear mixed model. (E) Kinetics of the ratio of *Esm1* expression intensity in epithelial cells over endothelial cells on whole lung sections collected at days 0, 1, 3 and 5 of ALI. A significantly higher ratio is observed in the LPS group (Φ = 0.003 by linear mixed model), especially at day 3 (*p* = 0.03 by two‐sided Mann‐Whitey test). (F) Variation of endocan blood levels during ALI. Significantly lower blood levels of endocan are observed in the LPS group (Φ = 0.009 by linear mixed model), especially at day 1 (*p* = 0.02 by two‐sided Mann Whitney test). Blood levels then progressively increase to reach values observed in the negative control group by day 5. (G) Correlation graph between endocan blood levels and the ratio of epithelial expression intensity over endothelial expression intensity in mice instilled with PBS and LPS. A negative correlation is obtained between these variables (*r* = −0.48, *p* = 0.008 by two‐sided Spearman test), suggesting that lower blood levels of endocan are associated with a lower fraction of pulmonary endothelial expression. *Esm1* absolute expression was assessed on whole lung sections by quantifying the *Esm1* signal trough fluorescence intensity and attributing it to either epithelial expression if colocalized with *Ccdc153* signal or endothelial expression otherwise. Expression intensity was defined as the ratio of absolute expression over the surface of area of interest. The ALI model was obtained by performing intratracheal instillation of LPS (2 mg/kg diluted in PBS 50 μL) in 8–12 weeks old C57BL/6 male mice. Negative control was obtained by intratracheal instillation of 50 μL of PBS in 8–12 weeks old C57BL/6 male mice. For all mice, general anaesthesia was performed through intraperitoneal injection of ketamine (75 mg/kg) and medetomidine (0.5 mg/kg) and reversed with a subcutaneous injection of 5 mg/kg of atipamezol. Euthanasia was performed through intraperitoneal injection of pentobarbital. Box plots show median, first and third quartiles, and whiskers at 1.5‐time interquartile range. ALI, acute lung injury; LPS, lipopolysaccharide; PBS, phosphate‐buffered saline.

Our results show that part of *Esm1* is intensely produced by the epithelial ciliated cells in mouse lungs, in accordance with the transcriptomic data. These results contrast with those of a study exploring the renal expression of endocan in mice, which identified endothelial cells as the quasi‐exclusive source of this molecule.^6^

In naïve mice, epithelial absolute expression is lower than the endothelial one, though a down‐regulation of the endothelial fraction of *Esm1* expression seems to be observed in ALI until day 5. These variations show some dissimilarities with the apparent increase observed in endocan blood levels from day 1 to 5 following LPS administration, implying a possible influence of extra‐pulmonary sources of expression of endocan.

The present results point out to the fact that we consistently observe trough different studies a major difference between the kinetics of endocan in human and mice. Indeed, in human, the blood levels of endocan are increased under pro‐inflammatory conditions, like sepsis, pneumonia or ARDS.^7^ On the other hand, in mice, we report in two previous publications from our group a decrease in blood levels of endocan following tracheal LPS‐induced ALI.^2^, ^3^ Therefore, the results shown in the present study seem consistent with our previous results in mice, though the discrepancy with the kinetics observed in human remains unexplained.

The type of ALI that was obtained through our model can be questioned as well. Indeed, by injecting directly LPS into the airways is likely to induce a model of primary ALI/ARDS. Therefore, it may not seem surprising that epithelial cells are more activated. There is still a possibility that in other types of secondary ARDS, the expression of endocan could be different and preferentially in the endothelial cells.

These results suggest that mouse models of ALI need refinement to be used for further studies regarding endocan. However, our results are exploratory and further studies to confirm them by other approaches, in vitro or in vivo, seem necessary.

## AUTHOR CONTRIBUTIONS

**Yoann Stordeur:** Conceptualization (supporting); formal analysis (lead); investigation (lead); methodology (supporting); writing – original draft (lead). **Sophie Salomé Desnoulez:** Formal analysis (supporting); writing – review and editing (supporting). **Patricia de Nadai:** Investigation (supporting); writing – review and editing (supporting). **Joanne Balsamelli:** Investigation (supporting). **Saliha Ait Yahia:** Investigation (supporting). **Anne Tsicopoulos:** Formal analysis (supporting); writing – review and editing (supporting). **Philippe Lassalle:** Writing – review and editing (supporting). **Alexandre Gaudet:** Conceptualization (lead); formal analysis (supporting); methodology (lead); supervision (lead); validation (lead); writing – review and editing (lead).

## CONFLICT OF INTEREST STATEMENT

The authors have disclosed that they do not have any conflicts of interest.

## Supporting information


Table S1.

## Data Availability

The data that support the findings of this study are available from the corresponding author upon reasonable request.
